# Assessing the consequences of prolonged usage of disposable face masks

**DOI:** 10.1038/s41598-022-20692-9

**Published:** 2022-10-07

**Authors:** Alessio Buzzin, Guillem Domènech-Gil, Elena Fraschetti, Ennio Giovine, Donatella Puglisi, Domenico Caputo

**Affiliations:** 1 Department of Information Engineering, Electronics and Telecommunications, Sapienza University of Rome, via Eudossiana, 18 00184 Rome, Italy; 2 Department of Physics, Chemistry and Biology, Sensor and Actuator Systems Division, Linköping University, Campus Valla, 581 83 Linköping, Sweden; 3 Institute for Photonics and Nanotechnologies, IFN - CNR, Via Cineto Romano 42, 00156 Rome, Italy

**Keywords:** Scanning electron microscopy, Electrical and electronic engineering, Quality of life

## Abstract

Due to the SARS-CoV-2 outbreak, wearing a disposable face mask has become a worldwide daily routine, not only for medical operators or specialized personnel, but also for common people. Notwithstanding the undeniable positive effect in reducing the risk of virus transmission, it is important to understand if a prolonged usage of the same face mask can have effectiveness on filtering capability and potential health consequences. To this aim, we present three investigations. A survey, carried out in central Italy, offers an overview of the distorted public awareness of face mask usage. A functional study shows how prolonged wearing leads to substantial drops in humid air filtration efficiency. Finally, a morphological analysis reports the proliferation of fungal or bacteria colonies inside an improperly used mask. Our study highlights therefore that wearing a face mask is really beneficial only if it is used correctly.

## Introduction

The world’s countries faced the SARS-CoV-2 pandemic outbreak with different attitudes and diversified national control strategies^1^. Governments’ guidelines and public awareness in each country differ and are constantly updated as new SARS-CoV-2 spreading dynamics and genetic mutations^2,3^ are discovered. For instance, Italy was one of the first and most affected countries by COVID-19 in the pandemic’s early stages and the first to order a nationwide lockdown on March 11, 2020^4^: timely and strict emergency measures, including the mandatory use of face masks for everyone both indoors and outdoors, were introduced to mitigate the spread of the virus and reduce the risk of infection. In contrast, countries from Northern Europe had a milder threat perception and have not adopted emergency measures to a similar extent^5^. At the time of writing this paper, a large part of the world population considers wearing a face mask as a normal daily routine^6,7^, which has a psychological, social, political, and cultural impact^8^. On the one hand, this new habit, together with other hygiene practices^9^, was demonstrated to be a key element in containing and slowing down the spread of the virus^10,11^. On the other hand, the urgency caused by the rapid escalation of the pandemic has led governments to react quickly and force their citizens to adopt these medical protection equipment^12^ without, however, providing an in-depth and comprehensive insight of their distinctive features, correct practices, proper wearing techniques, and disposal methods. The result has been a partial, inadequate, or even distorted awareness of the general public about what a face mask is, how to use it correctly, and why^13,14^. This fact led to misbehaviors in the population which may cause adverse health effects in both the short and long term and, therefore, needs to be recognized by health organizations and legislators as soon as possible^15,16^.

Medical face masks are commonly referred to as surgical or procedural masks, with performance features in terms of filtration, breathability, and fluid penetration resistance which are tested according to a set of standardized procedures and are designed to fit specific usage in medical environment^17^. Filtering facepiece respirators (FFRs) are disposable devices subject to other sets of regulatory standards, corresponding to different physical properties and performance characteristics. They are commonly used not only for protection against aerosols but also dust and microparticles, and are available in different classes depending on their filtering efficiency.

For example, in terms of filtration, medical masks can filter 3 µm-sized droplets, whereas FFRs can filter 0.075 µm-sized solid particles; European “FFP2” FFRs are able to filter at least 94% solid NaCl particles and oil droplets, whereas “N95” FFRs from US can filter at least 95% NaCl particles^18,19^. Moreover, the five layers of filtration material and the FFR shape, which seals around the wearer’s nose and mouth, guarantee the filtration claimed by the corresponding standards, while medical masks, which have three filtration layers and an open shape, provide a naturally less performant and leaking structure. Last and least effective are the custom-made cloth masks^9,20^. With all these elements taken into consideration, appropriate use, storage, cleaning, and disposal are essential to ensure their effectiveness and to limit transmission risks, regardless of the mask type^9^.

It is widely known that, as the wearer breathes, talks, coughs or sneezes, water vapor and droplets are produced, which can carry viruses and transmit infections (as in the case of SARS-CoV-2)^21–31^. By examining the effects of common practices on disposable face masks and the device deterioration over time, here we demonstrate why a correct usage is important to prevent potentially adverse health effects. In this framework, we report two evidences of possible face masks’ deterioration: (1) functional deterioration related to a decrease in humid air filtering capability; (2) morphological deterioration related to a mechanical degradation of the face mask layers. As a first step, we conducted a survey to investigate people’s habits in wearing disposable face masks. Then, we carried out a functional analysis on the filtering properties of FFRs and, afterwards, a morphological inspection of single layers of FFRs. For this purposes, disposable face masks were worn for extended periods of time (up to 24 h) and tested before and after usage. In particular, the 24-h used face masks were wore for three 8-h periods in consecutive separated days and dried inside an airtight box before analysing them. For our study, we used FFRs, as they provide better performance with respect to medical masks.

This study offers a better understanding of face masks characteristics and its appropriate use, and warns about the effects and potential risks of prolonged wearing. The information here presented can help to raise awareness among the general population and induce an improved, more adequate usage of such a crucial personal protective equipment.

## Results

### Face masks wearing awareness survey

**Figure 1:**
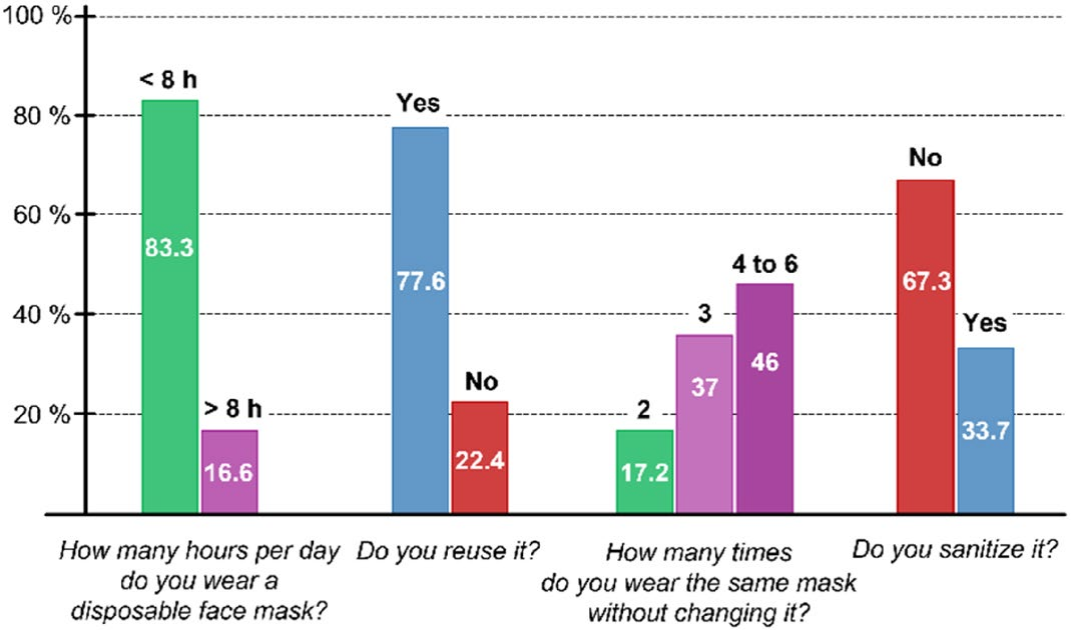
Face masks wearing awareness survey.

Four simple questions were asked to address people’s habits in terms of face mask use, with particular focus on wearing time and mask re-use. Figure 1 shows the results of our survey. They are presented without distinguishing gender, since we did not observe any significant differences between women and men practices. Based on the received answers, 83.3% of the survey respondents make a daily uninterrupted use of the face mask below 8 h, while 16.6% exceed 8 h every day. Only 22.4% of the participants confirmed to dispose the single-use mask after a few hours and wear a new one. However, 77.6% of the sample reported that, although they wear single-use face masks, they tend to reuse them multiple times. More specifically, 17.2% of this group reuses the same mask two times, whereas 82.8% declares to reuse it between three and six times (37.0% three times, 46.0% four to six times). Finally, 33.3% of the respondents state to regularly sanitize their mask after the first use (with alcohol-based products), while 67.3% do not care about sanitizing their masks at all.

### Functional characterization of disposable face masks: long-term relative humidity transmittance assessment

**Figure 2:**
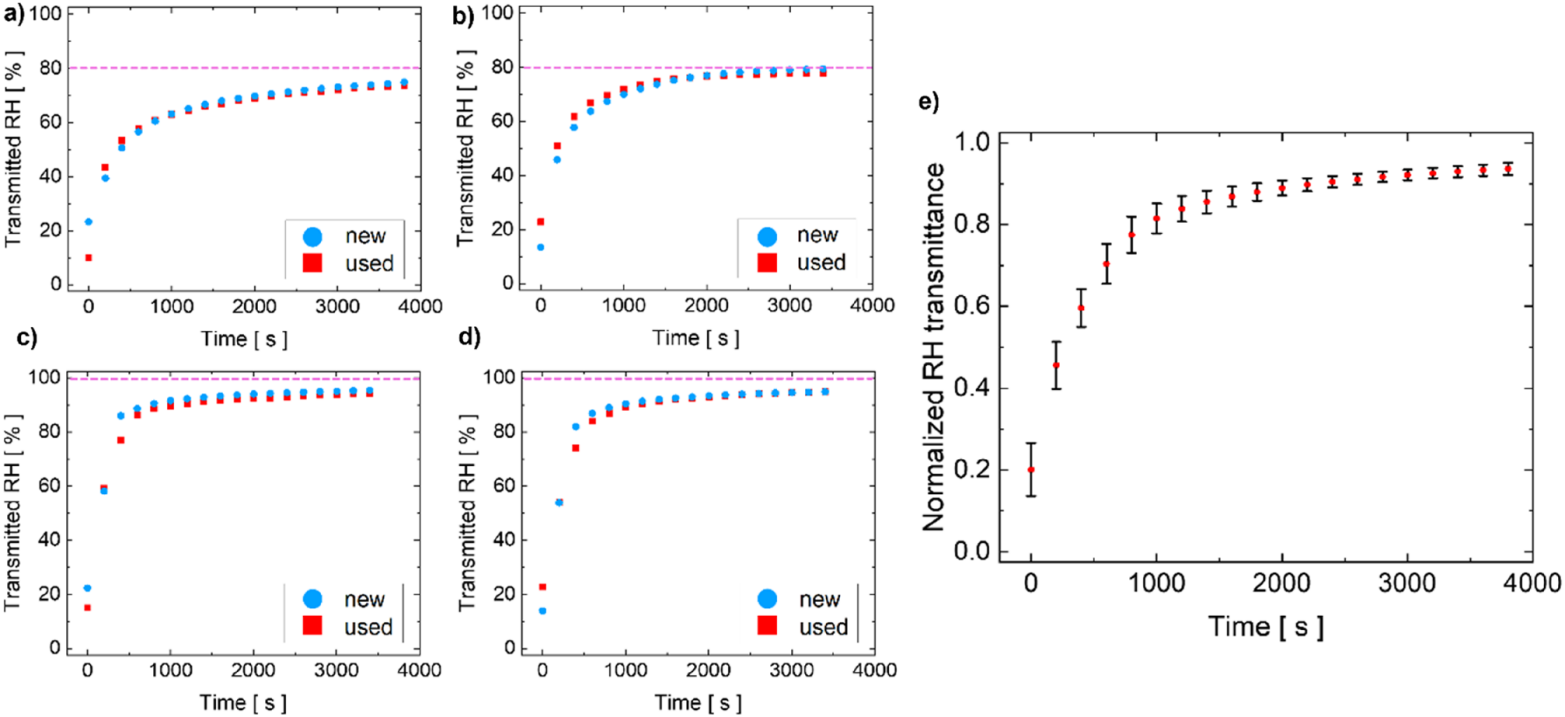
Time evolution of relative humidity transmitted through a new and 24-h used face mask in two experiments: gas source set to 80% RH diluted in synthetic air (pink dotted line) outwards (**a**) and inwards (**b**); gas source set to 100% RH diluted in pure nitrogen (pink dotted line) outwards (**c**) and inwards (**d**); mean values and standard errors of the eight normalized transmittances (**e**).

Even though not specifically related to virus transmission through water vapor and droplets^32,33^, measuring the relative humidity (RH) transmittance can be a simple, rapid and worst case method for testing the performance degradation of FFRs. We examined RH transmittance of new and used FFRs, both inwards and outwards, with two humid gas sources (Fig. 2): 80% RH in synthetic air (SA); and 100% RH in pure (5 N) nitrogen (N_2_). In all cases, the filtering capabilities monotonically decrease with time. The transmitted RH increases rapidly from about 20% to 60%—80% (depending on the source). Then, the transmitted RH saturates, with the source value acting as horizontal asymptote. By normalizing the eight curves (as Fig. 2e shows), we were able to observe a behaviour over time that can be mathematically well reproduced (R-squared equal to 0.99) through the hyperbolic function T(t):

$$ T\left( t \right) = \frac{at}{{b + t}} $$

where T represents the RH transmittance at the Y-axis, a and b are the two fitting parameters (here equal to 0.99 and 117.33, respectively) and t is the time at the X-axis.

### Morphological characterization

**Figure 3:**
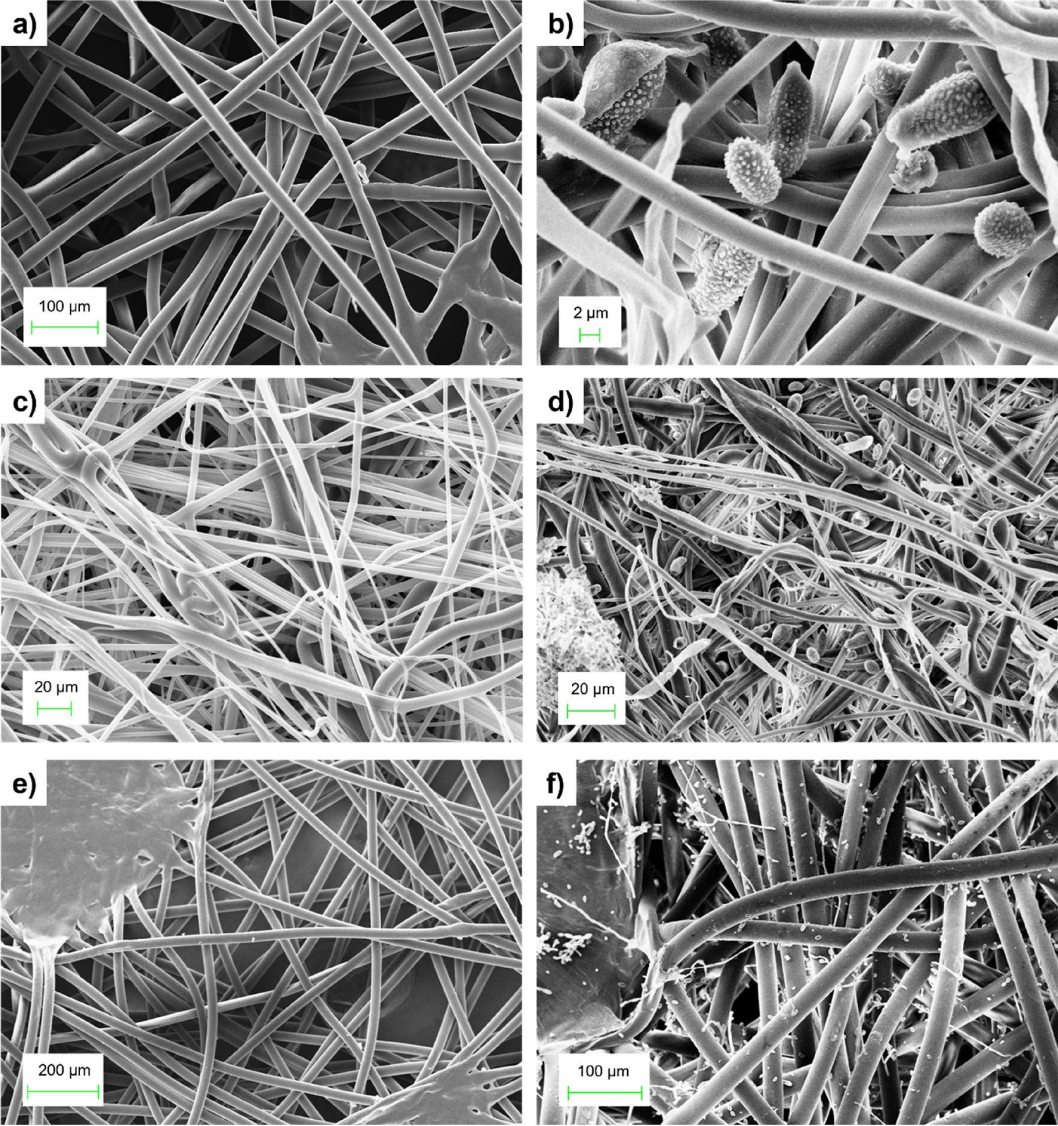
Layer-by-layer SEM comparison of a FFP2 facemask, before and after a 24-h usage: (**a**) Inner layer, before wearing, (**b**) Inner layer, after 24 h, (**c**) Middle layer, before wearing. (**d**) Middle layer, after 24 h, (**e**) Outer layer, before wearing, and (**f**) Outer layer, after 24 h.

**Figure 4:**
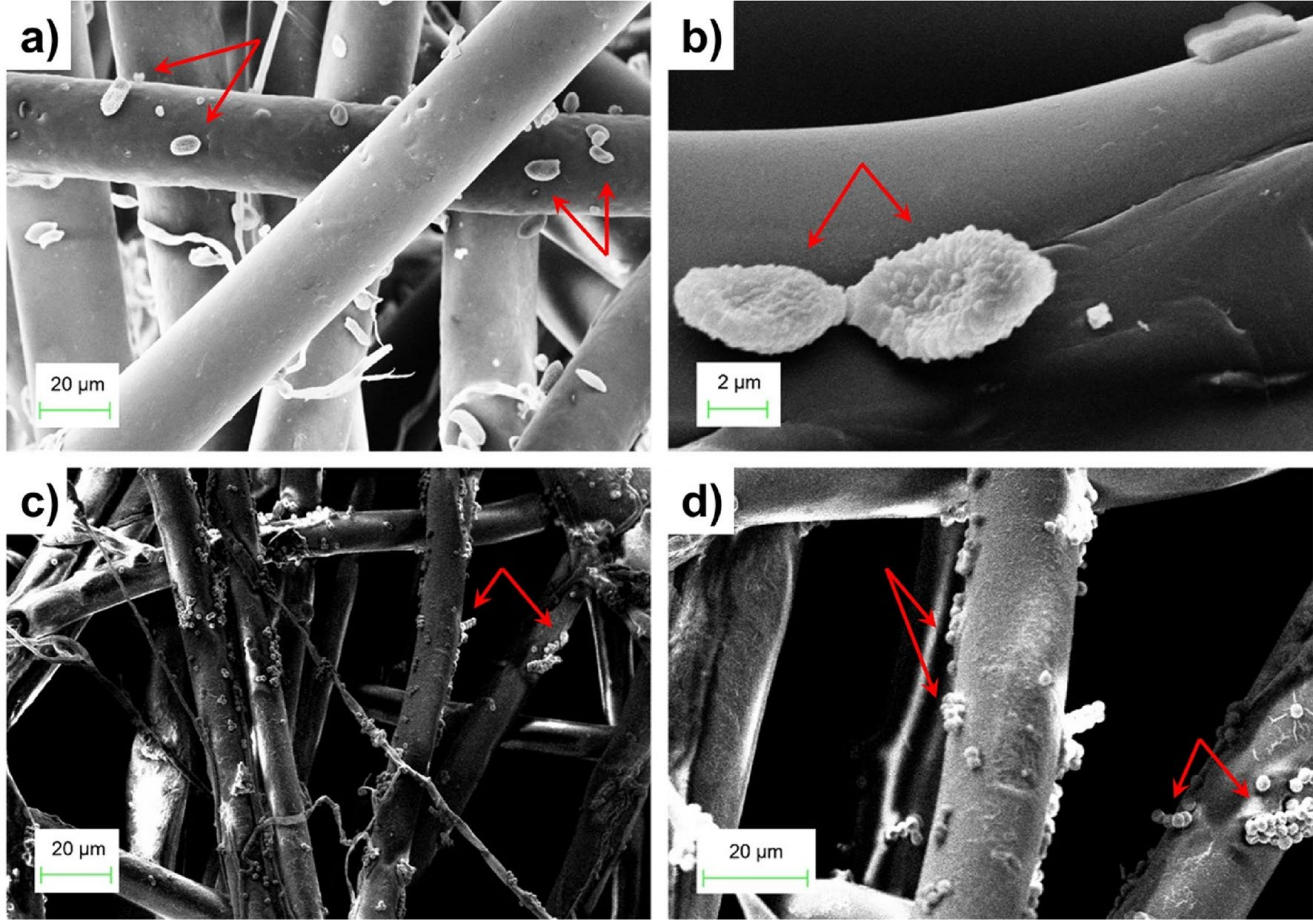
SEM insight: filaments of 24-h used FFP2 facemasks with fungi and spore formations.

Three of the five filtering layers of both new and used FFP2 face masks were inspected using a scanning electron microscope (SEM): the inner layer (in contact with the wearer’s skin), the outer layer (in contact with the external environment) and the central layer. As the layer-by-layer comparison shows (Fig. 3), no significant changes in morphological features can be observed before and after use. This confirms the results in Fig. 2, where new and used masks display the same filtering behavior. However, looking to Fig. 4, which shows a closer insight of the SEM images, fungi and fungal spore formations^34^ can be clearly seen in the textures after 24 h of use. These spores (indicated by red arrows) could reveal the presence of Conidia of Aspergillus as well as spore belonging to the family of Pucciniaceae^35^ or bacteria of the genus Staphylococcus.

## Discussion

The collected survey data highlight the lack of awareness of the basic principles of single-use face masks, despite stringent recommendations and legal actions adopted during an advanced stage of the pandemic event. Assuming six to eight working/studying hours per day as average, a significant part of the population wears the same single-use face mask up to a week, without any concerns about the equipment degradation, and a potential exposure to unnecessary health risks. This confirms that government rules do not automatically lead to a proper face masks usage by the general population.

According to our RH transmittance tests, the fast and significant drop in humid air filtering capability demonstrates a rapid functional degradation not specifically related to bioaerosols transmission. Moreover, no significant differences were observed for new and used masks in both directions: all the collected curves have a hyperbolical mean behavior, with a standard error that never exceeds 7% (absolute percentage) in the first minutes, and drops below 2% (absolute percentage) at the saturation stage. This strongly states that there are no substantial changes in the structure of the filter layers of an overly used mask with respect to a brand new one, but also demonstrates a noticeable humid air filtering degradation during the first 60 min of use.

As an additional note, when the internal environment of an FFR retains humidity relative to human breath, moisture and temperature also increase, up to 100% RH after 60 min of use^36^. These factors generally affect both health care professionals and common people suffering from severe discomfort during prolonged use of FFRs^37,38^. Therefore, the practice of wearing an FFR disposable face mask for excessive time is not only uncomfortable but can affect the health of the wearer.

From a morphological point of view, our results show that using a disposable face mask for several hours, without proper disposal or cleansing, may lead to collecting, growing, and cumulating inorganic as well as organic matter. This observation adds another noteworthy factor to the increasing discomfort and the significant drop in filtering performance. Moreover, our pictures confirm the assertions of previously published papers regarding bacterial formations^39^, providing new meaningful elements on this subject. The combined elements of high RH and relatively high temperature inside the mask, could create a microclimate within the face mask, including the growth of fungal and bacterial colonies. This fungi can efficiently colonize hosts with a compromised immune system, causing various forms of allergies. Normally, human hosts can manifest severe allergic reactions which can also result in lung damage. The primary site of infection is the lung. However, in the rarest but most severe cases, spreading to other organs and to the brain may also occur^40^.

Our reported data confirm the urgent need to inform the general public about the importance of wearing single-use face masks for shorter periods of time with respect to the current common practice, replacing them more frequently or, alternatively, sanitizing them properly. This is imperative especially in closed environments, where heat, humidity and CO_2_ produced by human breath, as well as the morphological nature of the mask, create a breeding ground for many kinds of life forms. However, disinfection or sterilization methods should be treated with caution, as they must guarantee effectiveness against SARS-CoV-2, that the mask is not damaged and does not lose its filtration capacity, and that they are not harmful to the person wearing the mask^41^.

As a conclusion we can say that, while it is evident and commonly accepted that wearing a face mask is very important for reducing the virus spread, especially in circumstances where proper ventilation and social distancing cannot be guaranteed, our study underlines that wearing a face mask is really beneficial only if it is used correctly. This latter aspect is still not well known or applied. The use of a disposable face mask should therefore be precisely defined and people should observe such rules not only to protect themselves from the risk of a viral infection but also to prevent other potential health problems.

## Methods

### Survey

The anonymity-friendly questionnaire was set out in line with the data minimization principle contained in the Guidelines of Processing Personal Data to Perform Customer Satisfaction Surveys in the Health Care Sector, published in Italy’s Official Journal No. 120 of 25 May 2011^42^. In particular, by following the last paragraph of point 4.1 of the above regulation, authors stated that the anonymity-friendly questionnaire was made following the considerations listed in the first and the second paragraphs of the same point 4.1, and therefore the processing of the relevant information does not fall under the scope of application of personal data protection and does not involve any ethical issues. Indeed, the survey’s scope and objective were defined in such a way that the information collected via the questionnaire did not contain any sensitive data, minimized the processing of users’ personal data, and was gathered in a way that the data subjects are not identifiable under any circumstances. Basing on these considerations, authors determined that it was not necessary to consult an ethics committee to obtain ethical approval waiver for survey research.

**Figure 5:**
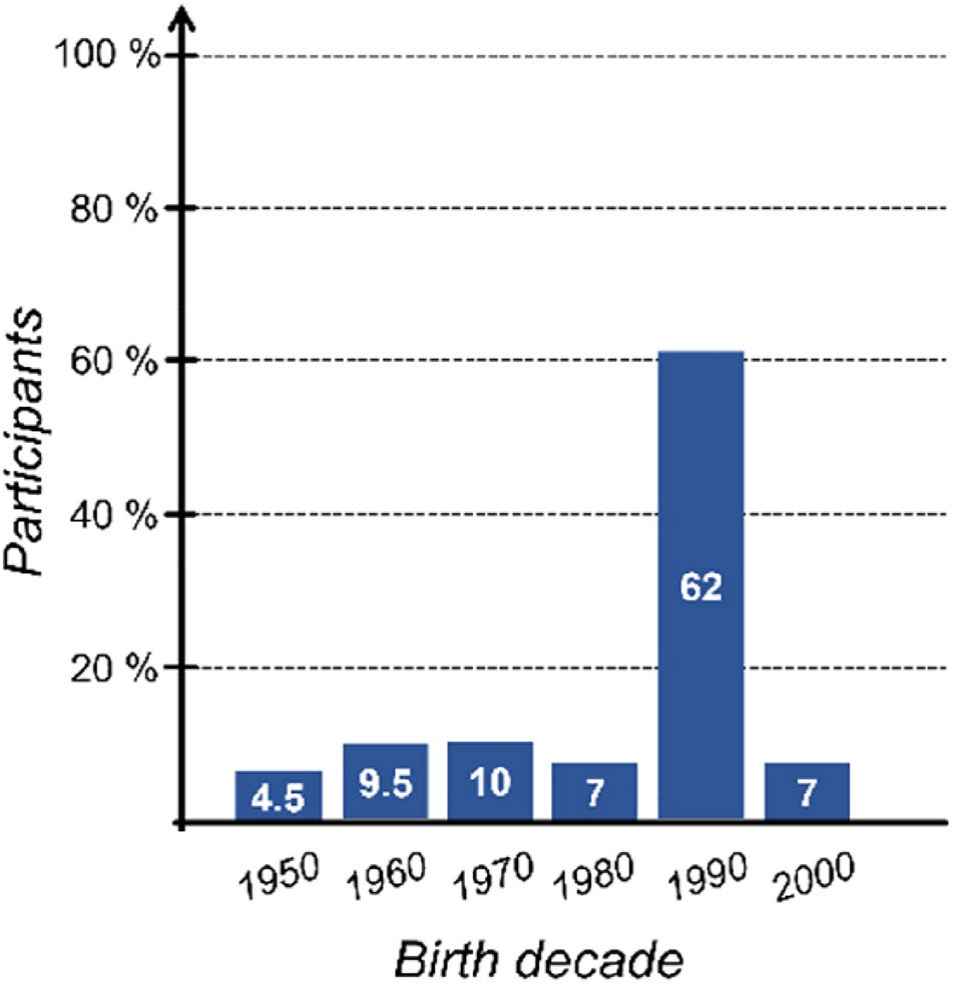
Survey participant classified by age.

The survey involved 190 participants, equally distributed between women and men, from 18 to 61 years old. We asked them to answer four questions via an anonymous Google form in accordance with relevant guidelines and regulations. The informed consent was obtained from all subjects. During the survey no experimental protocols were performed on the subjects. The observation area was the Italian capital city, Rome, and its surroundings. Participants born in the 1990s (21–31 years old) represented the largest age group, 62% of the total sample; the ones born in the 1970s and 1960s contributed to 10% and 9.5% of the sample, respectively; the ones born in the 1980s and 2000s contributed both with 7% of the sample, while 4.5% of the sample was represented by people born in the 1950s (see Fig. 5).

### Relative humidity tests

**Figure 6:**
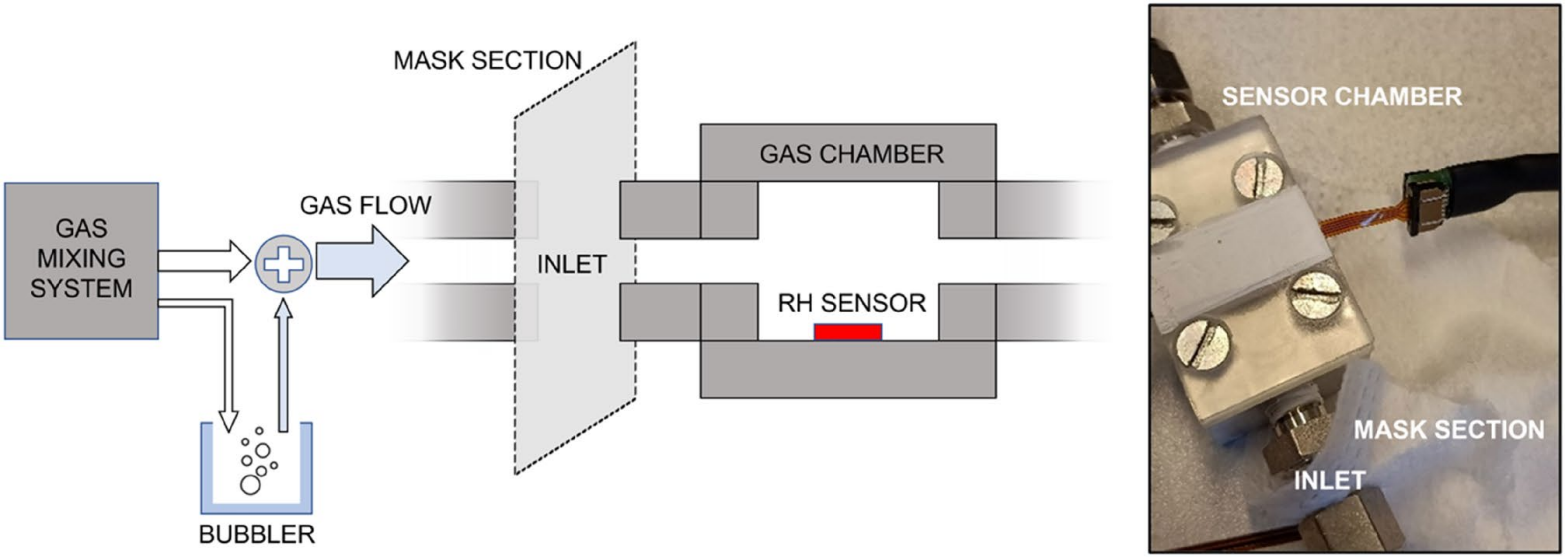
Relative humidity measurement setup scheme and picture of the gas test chamber with a face mask piece at the inlet, 3D-printed lid, and SHTC1 sensor during a measurement run.

The RH transmittance tests were performed using a commercially available digital humidity sensor, SHTC1 (Sensirion AG, Switzerland), which covers a measurement range from 0%RH to 100%RH with an accuracy of ± 3% in the range 20–80%RH and ± 4% in the ranges 0–20%RH and 80–100%RH, at ambient temperature (20 °C). The SHTC1 sensor was mounted in a custom-made stainless-steel chamber of about 3 mL volume, and enclosed with a low-cost, disposable lid, designed using a Formlabs Form 3 SLA 3D Printer (Fig. 6). Polytetrafluoroethylene (PTFE, trade name Teflon®), a highly hydrophobic and chemically inert material, was used to cover the plastic lid and to seal every interspace, opening and transition between regions: as a result, airtightness was assured, any unwanted chemical interactions were minimized, and any interference, adsorption, condensation or emission from/on the lid material were discarded.

The inlet of the gas chamber was connected to a gas mixing system with several Bronkhorst Mass-Flow Controllers, that have an accuracy between ± 0.5 and ± 2% of the total flow, driven by a custom-made software. Humidity was added by deviating a part of the gas flow through a bubbler. Sections of FFP2 face masks were tightened at the inlet of the gas chamber, between the humid gas flow source and the sensor’s region (Fig. 6). The measurements were performed from the inner to the external part of the face mask and vice versa, both for new and 24-h used face masks.

Every measurement run was carried out at a known RH concentration by pre-calibration of the sensor without the face mask. A total flow of 100 mL/min was kept constant for the whole campaign. Two configurations were chosen: 80%RH diluted in SA and 100%RH diluted in pure (5 N) nitrogen. Every measurement run had a duration of one hour. A 10-min flow of dry SA (< 5%RH) was provided between runs to decrease the RH concentration inside the gas chamber down to ambient temperature levels. Before each run, the airtightness of the complete setup was checked with a flow meter to guarantee comparable and reproducible measurements.

### Scanning electron microscope investigation

**Figure 7:**
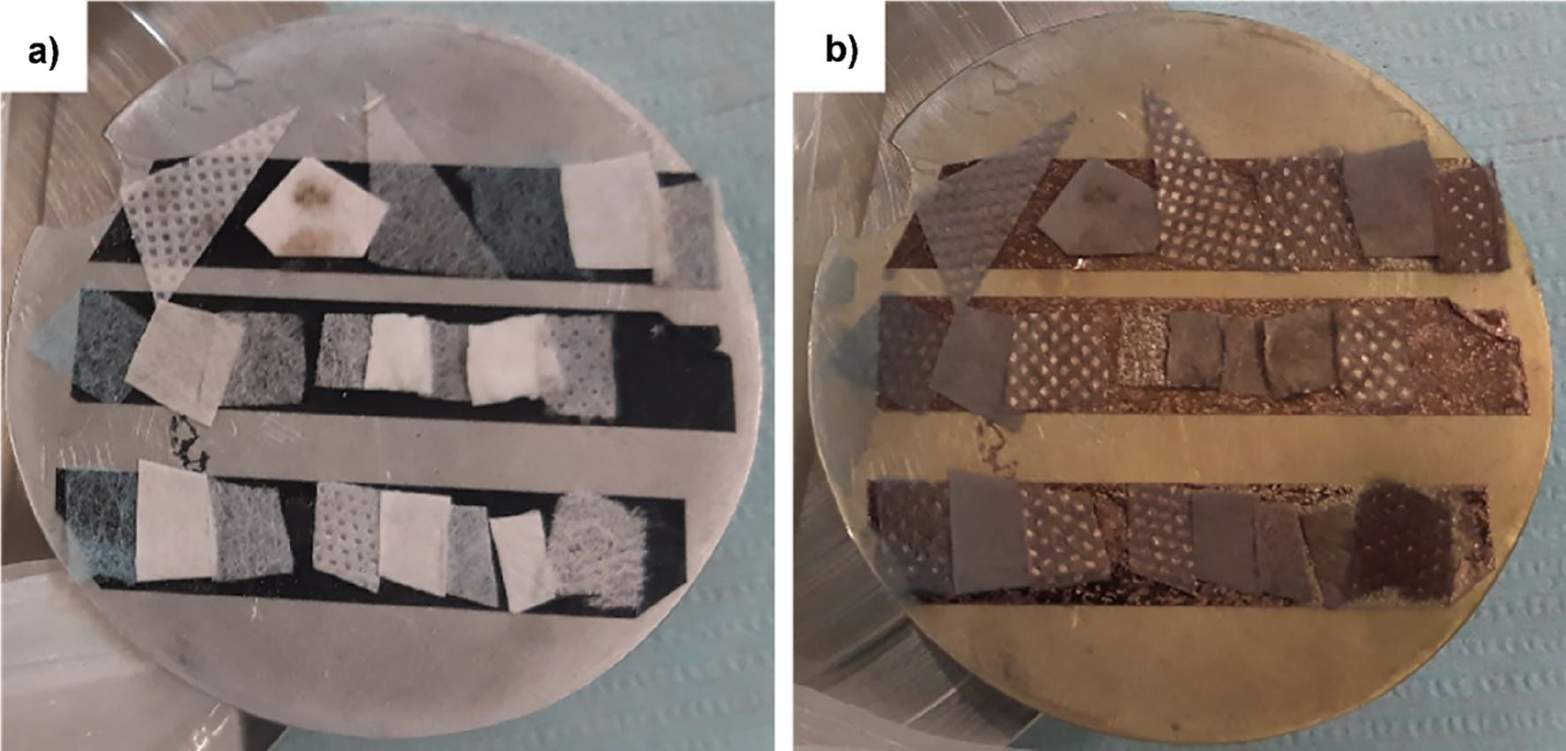
Face masks samples taped to a metallic stub before (**a**) and after (**b**) the gold sputtering as preparation for the SEM inspection.

For the reported SEM inspections, samples of the new and used FFP2 masks were cut from the central part, which is the most affected by the breath of the wearer’s mouth and nose. Each sample was then separated in its filtering layers and placed on a metallic stub to put inside the SEM vacuum chamber. Figure 7 shows samples of the masks’ separated filtering layers on the metallic stub during preparation for the SEM inspections. To allow a proper SEM visualization of the investigated areas, the samples were sputter-covered with a thin layer (5 to 10 nm) of gold. Moreover, an electrically conductive bi-adhesive tape (the three black stripes in Fig. 7) was used to secure the samples to the substrate and, at the same time, avoid the charge overloading phenomenon.

## Ethics approval

The anonymity-friendly questionnaire was set out in line with the data minimization principle contained in the Guidelines of Processing Personal Data to Perform Customer Satisfaction Surveys in the Health Care Sector, published in Italy’s Official Journal No. 120 of 25 May 2011^42^. Indeed, he survey’s scope and objective were defined in such a way that the information collected via the questionnaire did not contain any sensitive data, minimized the processing of users’ personal data, and was gathered in a way that the data subjects are not identifiable under any circumstances. By following the last paragraph of point 4.1 of the above regulation, authors stated that the anonymity-friendly questionnaire was made following the considerations listed in the first and the second paragraphs of the same point 4.1, and therefore the processing of the relevant information does not fall under the scope of application of personal data protection and does not involve any ethical issues. In agreement with these considerations, authors determined that was not necessary to consult an ethics committee to obtain an ethical approval waiver for survey research.

## Data Availability

All the dataset used and analysed during the current study are available from the corresponding author on reasonable request.
